# Analysis of the temperature-dependent plastic deformation of single crystals of quinary, quaternary and ternary equiatomic high- and medium-entropy alloys of the Cr-Mn-Fe-Co-Ni system

**DOI:** 10.1080/14686996.2024.2376524

**Published:** 2024-07-08

**Authors:** Le Li, Zhenghao Chen, Seiko Tei, Yusuke Matsuo, Ryosuke Chiba, Koretaka Yuge, Haruyuki Inui, Easo P. George

**Affiliations:** a Department of Materials Science and Engineering, Kyoto University, Sakyo-ku, Kyoto, Japan; b Materials Science and Engineering Department, University of Tennessee, Knoxville, TN, USA; c Institute for Materials, Ruhr University Bochum, Bochum, Germany

**Keywords:** High-entropy alloys, single crystals, critical resolved shear stress, thermal activation, mean-square atomic displacement

## Abstract

Temperature-dependent plastic deformation behaviors of single crystals of quaternary and ternary equiatomic medium-entropy alloys (MEAs) belonging to the Cr-Mn-Fe-Co-Ni system were investigated in compression at temperatures in the range 9 K to 1373 K. Their critical resolved shear stresses (CRSSs) increase with decreasing temperature below room temperature. There is also a dulling of the temperature dependence of CRSS below 77 K due to dislocation inertial effects that we attribute to a decrease in the phonon drag coefficient. These behaviors were compared with those of previously investigated single crystals of the equiatomic Cr-Co-Ni and Cr-Fe-Co-Ni MEAs, and the equiatomic Cr-Mn-Fe-Co-Ni high-entropy alloy (HEA). The temperature dependence of CRSS and the apparent activation volumes below room temperature can be well described by conventional thermal activation theories of face-centered cubic (FCC) alloys. Above 673 K, there is a small increase in CRSS, which we believe is due to elastic interactions between solutes and mobile dislocations, the so-called Portevin-Le Chatelier (PL) effect. The CRSS at 0 K was obtained by extrapolation of fitted CRSS vs. temperature curves and compared with predictions from solid solution strengthening models of HEA and MEAs.

## IMPACT STATEMENT

The novelty of our work entitled ‘*Analysis of the temperature-dependent plastic deformation of single crystals of quinary, quaternary and ternary equiatomic high- and medium-entropy alloys of the Cr-Mn-Fe-Co-Ni system*’ can be summarized as follows:

The temperature dependences of CRSS were experimentally deduced from bulk single crystals of the six MEAs for the first time, so that fair comparison among the FCC HEA/MEAs is made.

## GRAPHICAL ABSTRACT

**Figure:**
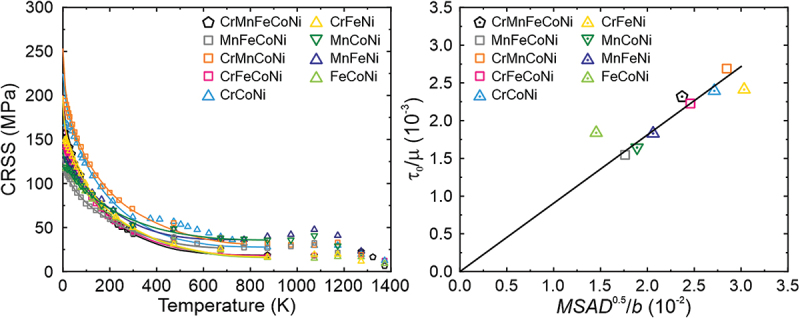

## 1. Introduction

A new class of multi-component concentrated alloys, often referred to as HEAs and MEAs, has aroused considerable interest in materials science and engineering [1–5]. Among these, single-phase, equiatomic, face-centered cubic (FCC) alloys belonging to the Cr-Mn-Fe-Co-Ni system although extensively investigated [6–10] remain scientifically interesting because they can help us better understand from a fundamental viewpoint structure-composition relationships and elementary mechanisms of deformation and failure in concentrated solid-solutions [11–14]. Some early reports speculated that HEAs and MEAs might become stronger as the number of constituent elements increased, but it is now generally accepted that solid solution strengthening (SSS) arises from the specific combinations of constituent elements rather than their sheer number [15,16]. The equiatomic quaternary and ternary MEAs derived from the Cr-Mn-Fe-Co-Ni system serve as useful prototypes to scientifically investigate the variation in mechanical properties as a function of element combinations by experiments and theoretical calculations. They also help us to understand how expensive constituents may be substituted by less expensive elements to achieve specific properties and a better cost-performance balance for practical applications. These factors motivate us to establish a sound understanding of the roles of constituent elements through the study of the equiatomic quaternary and ternary MEAs derived from the Cr-Mn-Fe-Co-Ni system.

Counting the quinary equiatomic Cr-Mn-Fe-Co-Ni HEA and its quaternary and ternary equiatomic MEA subsets, there are 16 alloys in all, 9 of which exhibit FCC single-phase microstructures after thermomechanical processing [17]. In our previous studies on single crystals of the equiatomic Cr-Mn-Fe-Co-Ni HEA, Cr-Fe-Co-Ni and Cr-Co-Ni MEAs [18–20], we have shown that their critical resolved shear stresses (CRSSs) at 0 K are consistent with the SSS model of Toda-Caraballo et al. [21] and also correlate linearly with the square root of MSAD (mean-square atomic displacement) [16,22]. In the case of the other six MEAs, however, although the temperature dependence of yield strength has been investigated in polycrystals [15,23], experimental studies on single crystals are lacking, despite the fact that some of them (such as the Cr-Mn-Co-Ni and Cr-Fe-Ni MEAs) exhibit higher or comparable yield strengths and tensile elongations in the polycrystalline form when compared with the equiatomic Cr-Mn-Fe-Co-Ni HEA [15,23].

In the present study, we investigate in compression the plastic deformation behavior of single crystals of quaternary and ternary equiatomic FCC MEAs belonging to the Cr-Mn-Fe-Co-Ni system at temperatures from 9 K to 1373 K. The CRSS values at 0 K, the temperature dependences of CRSS at low temperatures, the dulling of temperature dependence of CRSS at cryogenic temperatures, the small increase in CRSS at high temperatures and the apparent activation volumes for deformation are analyzed to elucidate the roles of each element. The results of the present study can help establish a benchmark for the fundamental plastic deformation behavior of FCC HEAs and MEAs.

## 2. Experimental procedures

Equiatomic quaternary and ternary MEAs belonging to the Cr-Mn-Fe-Co-Ni system (Table 1) were prepared as rods by arc melting high-purity (>99.9%) elemental metals in argon atmosphere. Single crystals were grown from these rods by either the optical floating-zone method (Mn-free alloys) or the Bridgman method (Mn-containing alloys), as described elsewhere [18,19]. The single crystals were annealed for 168 h at 1273 K or 1473 K (see Table 1), followed by water quenching. The chemical compositions after homogenization were measured on five different locations on the specimen surface by energy dispersive X-ray spectroscope equipped in a scanning electron microscope and the averaged values are tabulated in Table 1. After crystallographic orientations were determined by the Laue method, [$\bar{1}$23]-oriented specimens with gauge dimensions of 2 × 2 × 5 mm^3^ were cut by spark-machining for compression tests.

**Table 1.** Single crystal growth methods, homogenization temperatures and chemical compositions (in atomic percentage) after homogenization of the equiatomic alloys investigated in this study.

Equiatomic alloys	Single crystal growth method	Homogenization temperature (K)	Cr	Mn	Fe	Co	Ni
CrMnFeCoNi [18]	Bridgman	1473	20.39	19.81	19.79	19.95	20.06
MnFeCoNi	Bridgman	1273	–	26.61	24.51	24.34	24.54
CrMnCoNi	Bridgman	1273	26.91	24.74	–	24.90	23.45
MnFeNi	Bridgman	1273	–	32.09	35.61	–	32.30
MnCoNi	Bridgman	1273	–	32.09	–	34.44	33.47
CrFeCoNi [19]	Floating zone	1473	25.11	–	24.83	25.32	24.74
CrCoNi [20]	Floating zone	1473	33.65	–	–	33.22	33.13
CrFeNi	Floating zone	1473	33.82	–	34.03	–	32.15
FeCoNi	Floating zone	1473	–	–	33.44	33.49	33.07

Specimen surfaces were polished mechanically and then electrolytically with a solution of perchloric acid, n-butanol and methanol (1:2:7 by volume). Compression tests were conducted at temperatures between 9 K and 1373 K at an engineering strain rate of 1 × 10^−4^ s^−1^. Strain-rate jump tests were performed by suddenly changing the strain rate by an order of magnitude within the range of 1 × 10^−4^ s^−1^ to 1 × 10^−2^ s^−1^ below room temperature to measure the strain-rate sensitivity of flow stress. To minimize the strain hardening effect, strain-rate jump tests were conducted within the strain range of Stage I deformation (~10% plastic strain) of very small work hardening characteristics. Dislocation structures were observed by transmission electron microscopy (TEM) with a JEOL JEM-2000FX electron microscope.

Mean-square atomic displacements (MSADs) for the optimized SQS (special quasirandom structure) [24] configurations of equiatomic MEAs were estimated based on density functional theory (DFT) [25] by performing total energy calculations with the VASP code using projector-augmented wave method, with the exchange–correlation functional treated under the generalized-gradient approximation of Perdew-Burke-Ernzerhof (GGA-PBE). The details of the MSAD calculations of HEA and MEAs are described in our previous paper [20].

## 3. Results

### 3.1. Temperature dependence of CRSS

**Figure 1.**
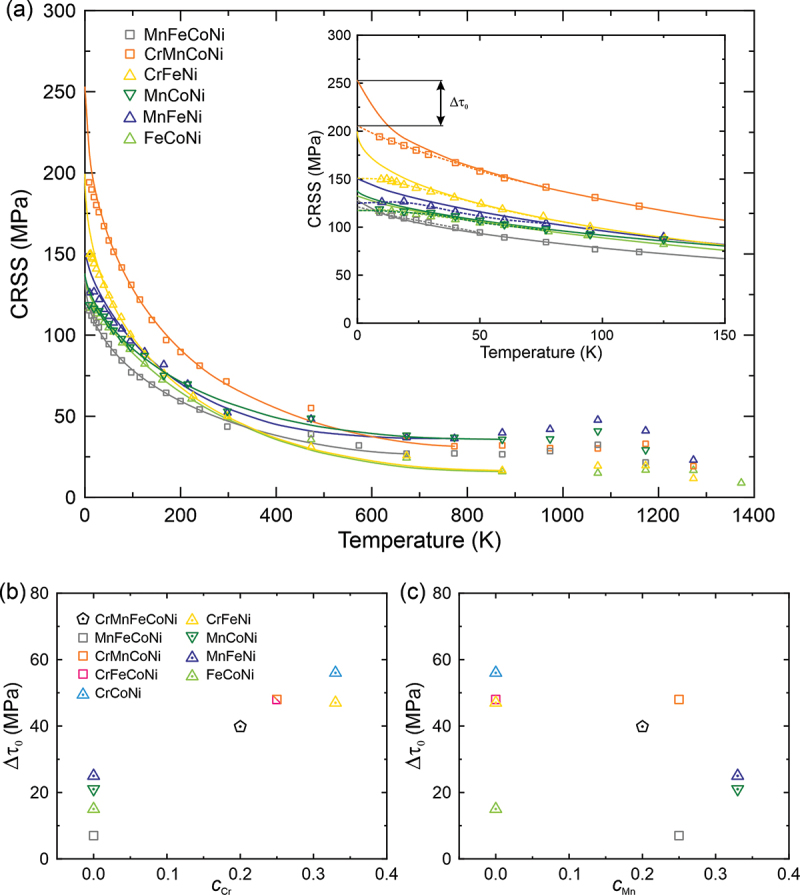
(a) Temperature dependence of CRSS for [123]-oriented single-crystals of equiatomic MEAs from 9 K to 1373 K in compression at a strain rate of 1×10^−4^ s^−1^. The inset shows a magnified view of the low-temperature region and an example of how the dulling of the CRSS (Δ*τ*_0_) due to inertial effects is measured. Δ*τ*_0_ is plotted as a function of Cr content in (b) and Mn content in (c).

From slip trace analysis, plastic flow was confirmed to initiate on (111)[$\bar{1}$01] in the two quaternary and four ternary MEAs listed in Table 1. This is the same slip system that operates in three other equiatomic alloys of this system, Cr-Mn-Fe-Co-Ni [18], Cr-Fe-Co-Ni [20] and Cr-Co-Ni [19]. CRSS values for (111)[$\bar{1}$01] slip obtained from the 0.2% offset yield stress and the Schmid factor of 0.467 for [$\bar{1}$23]-oriented single crystals are plotted in Fig. 1(a) as a function of temperature. Although the CRSS at a given temperature as well as the steepness of the temperature dependence of CRSS at low temperatures (below 300 K) varies significantly from alloy to alloy, the overall shapes of the temperature-dependent CRSS curves are similar. As shown in the inset of Fig. 1(a), CRSS rapidly increases with decreasing temperature below 300 K with some dulling of the temperature dependence occurring at the lower end (more on this later). At higher temperatures, the CRSS initially flattens but then increases moderately, peaking at 1000–1200 K depending on alloy. For the sake of completeness and to facilitate comparison, CRSS values previously obtained for equiatomic Cr-Mn-Fe-Co-Ni [18], Cr-Fe-Co-Ni [20] and Cr-Co-Ni [19] are provided in the Supplementary Material, Fig. S1.

CRSSs at 77 K (liquid nitrogen temperature, *τ*_LN_) and room temperature (*τ*_RT_) of the Cr-Mn-Fe-Co-Ni HEA and its MEA derivatives are tabulated in Table 2 along with their ratios (*τ*_LN_/*τ*_RT_). For single crystals of the MEAs investigated in the present study, the CRSS almost doubles when the temperature decreases from room temperature to 77 K, consistent with results obtained previously for single crystals of equiatomic Cr-Mn-Fe-Co-Ni [26], Cr-Fe-Co-Ni [27] and Cr-Co-Ni [28]. Also listed in this table are CRSS values estimated from polycrystals of these alloys obtained by dividing their yield stresses [15,16,23,29] by the Taylor factor of 3.06. Interestingly, the corresponding increase in CRSS with decreasing temperature is generally smaller for the polycrystalline alloys, excepting for the Cr-Fe-Ni MEA [23]. It is worth noting here that a relatively large grain size of 160 µm was tested in the case of the Cr-Mn-Fe-Co-Ni HEA [29] and Cr-Fe-Ni MEA [23], while the remaining MEAs had smaller grain sizes of 20 ~ 40 µm [15]. Grain-boundary (Hall-Petch) strengthening may overestimate the CRSS obtained from yield strengths of the latter alloys, which could account for the above discrepancy.

**Table 2.** CRSSs at 77 K (liquid nitrogen temperature) (*τ*_LN_) and room temperature (*τ*_RT_) of single crystals of the quinary, equiatomic HEA Cr-Mn-Fe-Co-Ni and its equiatomic quaternary and ternary MEAs. Data for single crystals of the equiatomic Cr-Mn-Fe-Co-Ni, Cr-Fe-Co-Ni and Cr-Co-Ni are from the indicated references. CRSS of polycrystals are all deduced from the yield strengths in Ref. [15] and other indicated references by dividing them by the Taylor factor of 3.06.

	Single crystals			Polycrystals^[15]^		
Equiatomic alloys	*τ*_LN_ (MPa)	*τ*_RT_ (MPa)	*τ*_LN_/*τ*_RT_	*τ*_LN_ (MPa)	*τ*_RT_ (MPa)	*τ*_LN_/*τ*_RT_
CrMnFeCoNi	109^[18]^	43^[18]^	2.53	126^[26]^	62^[26]^	2.03
	153^[26]^	56^[26]^	2.73			
MnFeCoNi	84	44	1.91	98	58	1.69
CrMnCoNi	142	71	2.00	164	92	1.77
CrFeCoNi	102^[20]^	44^[20]^	2.32	156	91	1.72
	96^[25]^	41^[25]^	2.34			
CrCoNi	133^[19]^	60^[19]^	2.22	168	99	1.69
	157^[27]^	78^[27]^	2.01			
CrFeNi	111	50	2.22	126^[23]^	57^[23]^	2.21
MnFeNi	104	52	2.00	120	75	1.61
MnCoNi	98	53	1.85	121	75	1.61
FeCoNi	95	49	1.94	112	70	1.60

### 3.2. CRSS at 0 K

To deduce the CRSS at 0 K, the experimentally obtained CRSS values were fitted with the following equation [30–32],

(1) $$\tau \left(T\right)=\tau_{\mathrm{ath}}+\tau_{\mathrm{th}}{\left[1-{\left(\frac{T}{T_{a}}\right)}^{\frac{1}{q}}\right]}^{\frac{1}{p}}$$

where *T* is the absolute temperature of the compression test, *T*_a_ is the athermal temperature above which the CRSS becomes temperature-independent, *τ*_ath_ is the athermal stress, i.e. CRSS at *T*_a_, *τ*_th_ is the thermal stress, and *p* and *q* are fitting parameters. For the fitting, we did not include CRSSs below the critical temperature (*T*_c_ in Table 3) at which dulling becomes discernable, to avoid interference from inertial effects as discussed in section 4.3. We also did not include data points above 873 K where a small increase in CRSS is observed with increasing temperature due to the PL effect as discussed later. The fitting results are tabulated in Table 3 and the fitted curves are shown in Fig. 1(a) and Fig. S1. The CRSS at 0 K (*τ*_0_) tends to increase with increasing Cr and Mn content, trends that we will attempt to rationalize later by taking into account the magnitudes of the averaged MSADs.

**Table 3.** The experimental 0 K CRSS values for single crystals of the quinary HEA and its derivative quaternary and ternary equiatomic alloys determined by fitting the CRSS-temperature curves to Eq. (1) from which the optimal parameters (1/*p* and 1/*q*) were obtained. Results for equiatomic Cr-Mn-Fe-Co-Ni, Cr-Fe-Co-Ni and Cr-Co-Ni are from previous studies [18–20].

	Single crystals								Polycrystals^[15,16,23,26]^		
Equiatomic alloys	*τ*_0_ (MPa)	Δ*τ*_0_ (MPa)	*T*_c_ (K)	*T*_a_ (K)	*τ*_ath_ (MPa)	*τ*_th_ (MPa)	1/*q*	1/*p*	*σ*_0_ (MPa)	1/*q*	1/*p*
CrMnFeCoNi	197	41	77	873	18.7	154.8	0.58	2.70	525	0.87	2.44
MnFeCoNi	124	7	50	773	27.1	96.5	0.67	2.19	375	0.86	2.17
CrMnCoNi	253	48	77	773	31.5	221.4	0.50	1.86	650	0.89	2.44
CrFeCoNi	200	48	77	873	18.1	181.8	0.50	2.20	641	0.85	2.70
CrCoNi	225	56	62	873	27.8	197.2	0.66	2.67	661	0.90	2.70
CrFeNi	199	47	77	873	16.6	182.6	0.51	1.99	626	0.73	2.62
MnFeNi	150	25	60	773	36.3	114.2	0.73	2.32	448	0.94	2.63
MnCoNi	138	21	77	873	35.8	102.0	0.67	2.27	466	0.91	2.70
FeCoNi	132	15	77	873	15.9	116.5	0.77	2.23	418	0.94	2.33

**Figure 2.**
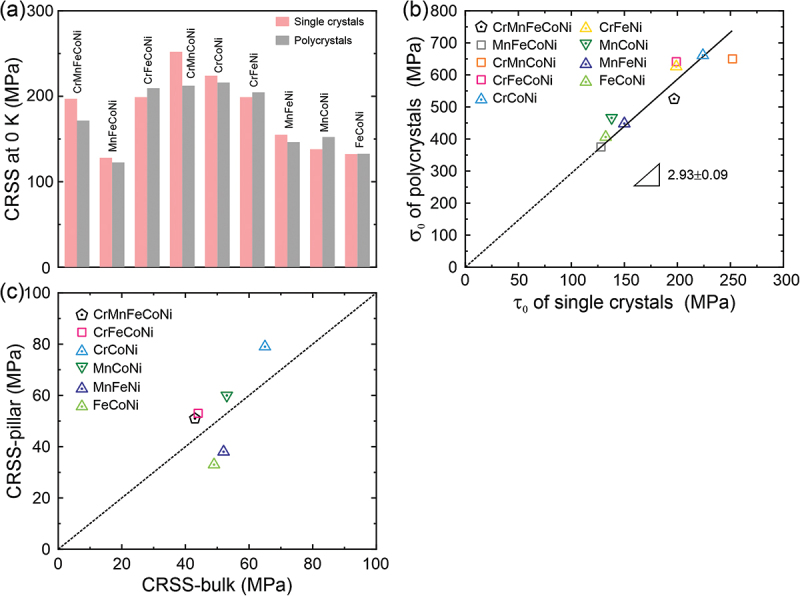
(a) The CRSS at 0 K obtained directly from single crystals compared with those obtained from polycrystals by dividing their yield strengths by the Taylor factor 3.06. (b) The 0 K yield strength of polycrystals ($\sigma_{0}$) plotted as a function of the 0 K CRSS ($\tau_{0}$) of single crystals. (c) Room-temperature CRSSs estimated from micropillar compression tests [34] compared with CRSSs obtained directly from bulk single crystals.

The fitting results for polycrystals of these alloys [15,16,23,29] are also listed in Table 3. Interestingly, while the ternary Cr-Co-Ni MEA is the strongest polycrystalline alloy [15], the quaternary Cr-Mn-Co-Ni MEA has the highest CRSS at 0 K among the single crystals. The *q* values of single crystals are generally larger than those of their polycrystalline counterparts, indicating a stronger temperature dependence of CRSS consistent with a higher *τ*_LN_/*τ*_RT_ ratio in single crystals. Interestingly, the *τ*_0_ of single crystals is comparable to the *σ*_0_ of polycrystals divided by the Taylor factor of 3.06 for most of the investigated alloys, as shown in Fig. 2(a). This correspondence is apparent also in Fig. 2(b) where the *σ*_0_ of polycrystals plotted as a function of the *τ*_0_ of single crystals exhibits a linear dependence with a slope of 2.93 ± 0.09, which is comparable to, but somewhat lower than, the Taylor factor of 3.06. As mentioned before, this slight discrepancy may be due to grain-boundary strengthening in the polycrystalline alloys.

Another way in which CRSSs can be determined is by compressing single-crystal micropillars and extrapolating the size-dependent CRSS values to pillar sizes of 20–30 µm to obtain ‘bulk’ CRSS values. This has been done previously [33,34]. Those extrapolated (micropillar) CRSSs are compared in Fig. 2(c) with CRSSs obtained directly by testing true bulk single crystals. Except for a couple of the alloys, the CRSSs estimated from single-crystal micropillars tend to be higher than those of bulk single-crystals (if they were the same, all data points would lie on the 45° line). This suggests the possibility of a remnant size effect (‘smaller is stronger’) even in pillars as large as 20–30 µm.

### 3.3. Dulling of temperature dependence of yield strength at cryogenic temperatures

A dulling (or decrease in the steepness) of the temperature dependence of CRSS has been observed in dilute FCC solid-solution alloys such as Cu-Al alloys [35], as well as in the Cr-Mn-Fe-Co-Ni HEA [36], Cr-Fe-Co-Ni [20] and Cr-Co-Ni [19] MEAs, and is believed to be due to inertial effects [35,37,38]. Visual examination of CRSS vs. *T* curves may not always be sensitive enough to detect dulling. A more sensitive technique for identifying the critical temperature (*T*_c_) below which inertial effects can affect dislocation motion involves determining the peak in the strain rate sensitivity of flow stress, as discussed in our previous work [19,20]. That is the procedure we used here for the six new single-crystal MEAs listed in Table 1. Engineering stress-strain curves of the Cr-Fe-Ni MEA deformed in compression at selected temperatures are shown in Fig. S2. As mentioned in section 3.1, magnified portions of the CRSS vs. *T* curves are shown in the inset of Fig. 1(a) (as well as the inset of supplemental Fig. S1). In these figures, the solid lines are fits of Eq. (1) to only those experimental data points that lie above *T*_c_, whereas the dashed lines go through all experimental data points. The identified critical temperatures (*T*_c_) are tabulated in Table 3, along with those determined previously for equiatomic Cr-Co-Ni and Cr-Fe-Co-Ni [19,20]. In the inset of Fig. 1(a), both the fitted solid curves and the dashed experimental curves have been extrapolated down to 0 K. For any given alloy, the CRSS difference between the solid and dashed curves at 0 K is considered to be the extent of dulling due to inertial effects ($\Delta \tau_{0}$ in the one example shown). This dulling ($\Delta \tau_{0}$) seems to be correlated with the Cr concentration (Fig. 1(b)) but not with the Mn concentration (Fig. 1(c)), despite these two elements having similarly large effective atomic radii and misfit volumes in the quinary, quaternary and ternary alloys of the Cr-Mn-Fe-Co-Ni system [9,39]. In contrast, the dulling of CRSS (Δ*τ*_0_) does not exhibit any definite correlation with the Fe, Co and Ni concentrations (see Fig. S3). Regardless, the manifestation of inertial effects in equiatomic alloys of the Cr-Mn-Fe-Co-Ni system is intriguing given theoretical calculations [38] and experiments on dilute Cu-Al alloys [35] that suggest these effects should become less prominent as the solute concentration increases and even disappear in highly concentrated solid-solutions.

### 3.4. Yield stress increase at elevated temperatures

**Figure 3.**
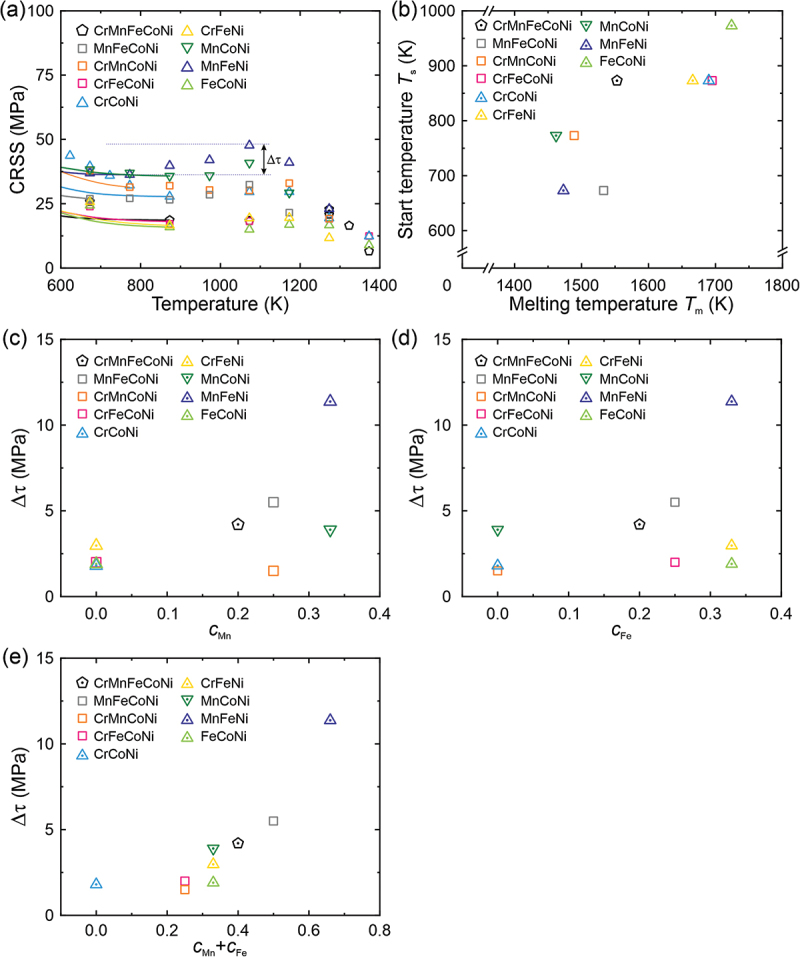
(a) The temperature dependence of CRSS of the investigated alloys from 600 K to 1373 K, showing the small increase in CRSS (Δ*τ*) due to the PLC effect. (b) The starting temperature of PLC is plotted against the melting temperature of the alloys. Δ*τ* is plotted as a function of Mn content in (c), Fe content in (d), and Mn plus Fe content in (e).

For all the investigated alloys, there is a slight increase of CRSS with increasing temperature between 673 K and 1273 K (Fig. 3(a)). Although the magnitude of CRSS increases as well as the temperature range where it occurs varies from alloy to alloy, the stress-strain curves in the relevant temperature range always exhibit serrations, as shown in stress-strain curves of Fig. S2 as an example for the Cr-Fe-Ni MEA. Thus, we attribute the increase in CRSS to the well-known Portevin-Le Chatelier (PL) effect. Table 4 lists the increase in CRSS (Δ*τ*), starting temperature *T*_s_ (defined as the temperature above which serrations occur in the stress-strain curves), peak stress temperature and melting temperature (*T*_m_) [15,23] of the investigated alloys. Tsai et al. [40] reported that the PL effect shifts to lower temperatures as the number of constituent elements increase in the Cr-Mn-Fe-Co-Ni system. However, we do not observe such a trend, see Table 4. Instead, the starting temperature of the PL effect (*T*_s_) roughly increases with the *T*_m_, as shown in Fig. 3(b). Note that the start of the PL effect occurs in the range *T*_*s*_ = 0.45 ~ 0.55 *T*_m_ (Table 4), which is also where atomic diffusion starts to become important, consistent with the general view that the mechanism of PL involves the cyclic pinning, breakaway, and re-pinning of dislocations by solutes that can diffuse at rates comparable to those of moving dislocations. Furthermore, the extent of CRSS increase due to the PL effect (Δ*τ*) shows a rough increase with increasing Mn and Fe contents (Fig. 3(c), 3(d)), and a clear increase with the sum of their concentrations (Fig. 3(e)), indicating that pinning by Mn and Fe probably plays a role in the PL effect.

**Table 4.** The increase in CRSS, starting temperature and peak temperature related to PL effect for equiatomic quinary Cr-Mn-Fe-Co-Ni HEA and its derivative quaternary and ternary alloys. The melting temperatures for these alloys are also listed.

	Δ*τ* (MPa)	*τ*_d_ (MPa)	*c*_Mn_	*c*_Fe_	Starting temperature (*T*_s_)(K)	Peak temperature (K)	Melting temperature^[15,23]^ (*T*_m_)(K)	*T*_s_/*T*_m_
CrMnFeCoNi	4.2	3.23	0.20	0.20	873	1273	1553	0.56
MnFeCoNi	5.5	6.78	0.25	0.25	673	1073	1533	0.44
CrMnCoNi	1.5	4.33	0.25	0.00	773	1173	1489	0.52
CrFeCoNi	2.0	0.26	0.00	0.25	873	1273	1695	0.52
CrCoNi	1.8	0.00	0.00	0.00	873	1173	1690	0.52
CrFeNi	3.0	0.10	0.00	0.33	873	1173	1666	0.52
MnFeNi	11.4	3.94	0.33	0.33	673	1073	1473	0.46
MnCoNi	3.9	7.28	0.33	0.00	773	1073	1462	0.53
FeCoNi	1.9	0.50	0.00	0.33	973	1173	1724	0.56

## 4. Discussion

### 4.1. Thermally activated glide of dislocations

According to Kocks et al. [30], the adjustable parameters *p* and *q* in Eq. (1) determine the glide resistance profile of obstacles. *p* is usually expressed as

(2) $$p=1-\frac{1}{n}\;\mathrm{with}\;\tau_{r}\propto \frac{1}{{\left(\Delta a\right)}^{n}}\left(\tau_{r}\ll \hat{\tau }\right)$$

where *τ*_r_ is the glide resistance, Δ*a* is the activation area, and $\hat{\tau }$ is the amplitude of glide resistance. According to Eq. (2), the ‘tail’ of the glide resistance of obstacles decays with the negative power of Δ*a*. The second parameter, *q*, describes the shape of the ‘top’ of the profile and is given by,

(3) $$q=1+\frac{1}{k}\;\mathrm{with}\;\tau_{r}=\hat{\tau }\left[1-{\left(\Delta a\right)}^{k}\right]$$

**Figure 4.**
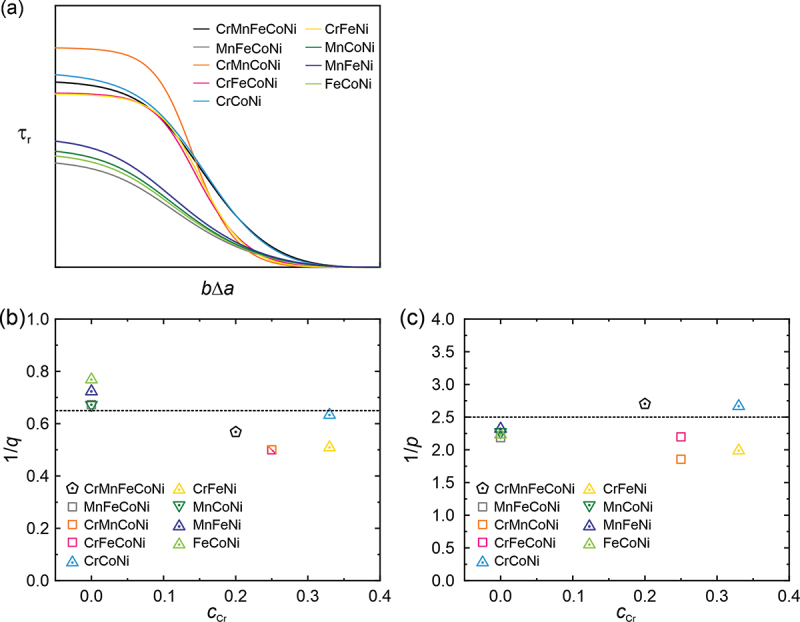
(a) The qualitative glide resistance ($\tau_{r}$) profiles of dislocations in different equiatomic alloys based on their respective *p* and *q* values. (b) 1/*q* and (c) 1/*p* values plotted against the atomic concentration of Cr in the different equiatomic alloys.

From the 1/*q* and 1/*p* values determined for single crystals in Table 3, the qualitative glide resistance profiles of the investigated alloys are calculated with Eqs. (2) and (3), and plotted in Fig. 4(a). Alloys containing Cr generally have larger *q* values than those containing no Cr (Fig. 4(b)), suggesting a slower decay in the glide resistance at the top for the Cr-containing alloys. Relatively speaking, among the Cr-containing alloys, equiatomic Cr-Fe-Co-Ni, Cr-Mn-Co-Ni, and Cr-Fe-Ni have somewhat smaller 1/*q* than the equiatomic Cr-Co-Ni and Cr-Mn-Fe-Co-Ni (Fig. 4(b)), but display the opposite trend in 1/*p* values (Fig. 4(c)). This suggests that in the equiatomic Cr-Mn-Fe-Co-Ni HEA and Cr-Co-Ni MEA, although the glide resistance decays relatively quickly at the top, it decays more slowly at the tail, as shown in Fig. 4(a). It is worth noting that the range of 1/*q* (0.50–0.76) and 1/*p* (2–2.7) of the present HEA and MEA single crystals is comparable to those (1/*q* = 0.67, 1/*p* = 2.17) of dilute Cu-Mn alloys (up to 7.6 at.% Mn) [32]. However, Leyson et al. [41] and Varvenne et al. [39] used values of 1/*q* = 0.67 and 1/*p* = 1 to model solid solution strengthening in FCC HEA and MEAs. This may be a reason for the difference in the measured and calculated CRSSs of equiatomic alloys of the Cr-Mn-Fe-Co-Ni system, in particular, at cryogenic temperatures [9].

**Figure 5.**
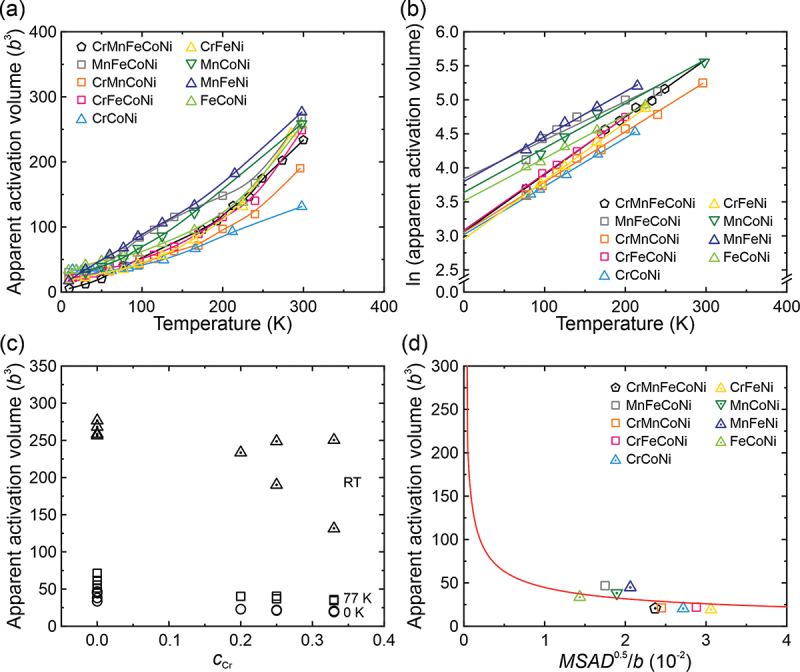
(a) Temperature dependence of apparent activation volume for deformation of single crystals of the HEA and MEAs and (b) the temperature-dependent apparent activation volumes fitted with Eq. (5). (c) Apparent activation volumes plotted as a function of Cr content at selected temperatures. (d) Apparent activation volumes of HEA and MEAs at 0 K plotted as a function of the square root of MSAD.

In solid-solution alloys, the strong glide resistance may lead to not only a significant temperature dependence of CRSS but also a lower apparent activation volume of dislocation motion. The apparent activation volume (*V*) can be estimated from the strain-rate ($\dot{\gamma }$) sensitivity of flow stress (*τ*) by the following conventional equation,

(4) $$V=k_{B}T{\left[\frac{\partial \ln\dot{\gamma }}{\partial \tau }\right]}_{T}$$

in which *k*_B_ is the Boltzmann constant. The calculated apparent activation volume of the investigated MEAs is plotted in Fig. 5(a) as a function of temperature, together with those of equiatomic Cr-Mn-Fe-Co-Ni [18], Cr-Fe-Co-Ni [20] and Cr-Co-Ni [19]. The temperature dependence of the apparent activation volume of FCC solid-solutions can be expressed by [42,43],

(5) $$V=V_{0}\exp\left(\frac{-mk_{B}T}{W_{0}}\right)$$

where *V*_0_ is the apparent activation volume at 0 K, *m* is a constant around 27 and *W*_0_ is a material-dependent constant. The experimentally deduced apparent activation volumes (Fig. 5(a)) were fitted with Eq. (5), excluding data points in the temperature range for the inertial effects, as shown in Fig. 5(b). The apparent activation volumes at 0 K, 77 K and room temperature are tabulated in Table 5 and plotted as the function of Cr content (*c*_Cr_) in Fig. 5(c). At cryogenic temperatures (0 and 77 K), the apparent activation volume generally decreases with increasing Cr concentration. This is consistent with the general view that the apparent activation volume is inversely correlated with the strength of the temperature dependence of CRSS in FCC solid-solution alloys.

**Table 5.** The apparent activation volumes at 0 K, 77 K (liquid nitrogen temperature, *V*_LN_) and room temperature for single crystals of the quinary HEA and its derivative quaternary and ternary equiatomic alloys.

	*c*_Cr_	*c*_Mn_	*V*_0_ (*b*^*3*^)	*V*_LN_ (*b*^3^)	*V*_RT_ (*b*^3^)
CrMnFeCoNi [18]	0.20	0.20	23	40	234
MnFeCoNi	0.00	0.25	47	62	268
CrMnCoNi	0.25	0.25	21	37	190
CrFeCoNi [20]	0.25	0.00	22	40	249
CrCoNi [19]	0.33	0.00	20	36	131
CrFeNi	0.33	0.00	19	34	251
MnFeNi	0.00	0.33	45	71	277
MnCoNi	0.00	0.33	38	51	259
FeCoNi	0.00	0.00	34	55	257

The 0 K apparent activation volumes of the alloys in the present study are found to be inversely proportional to the square root of MSAD, as shown in the bottom of Fig. 5(d). According to Butt et al. [44], the apparent activation volume at 0 K can be expressed as

(6) $$V_{0}=\frac{1}{4}b^{3}l^{2}{\left(\frac{\mu b^{3}}{Ec^{1/2}}\right)}^{1/2}$$

where *E* is the interaction energy between a dislocation segment and solutes, *c* is the solute concentration, *lb* denotes the distance traveled by a dislocation segment during an activated jump (*b* is the Burgers vector of a full dislocation), and *μ* is the shear modulus. The CRSS at 0 K is [44]

(7) $$\tau_{0}=\frac{4Ec^{1/2}}{lb^{3}}$$

Substituting Eq. (7) into (6), we obtain

(8) $$\frac{V_{0}}{b^{3}}=\frac{1}{2}l^{3/2}{\left(\frac{\tau_{0}}{\mu }\right)}^{-1/2}=\frac{1}{2}l^{3/2}C^{-1/2}{\left(\frac{\mathrm{MSA}D^{1/2}}{b}\right)}^{-1/2}$$

where *C* is the constant in the relationship between CRSS at 0 K and the square root of MSAD (as will be discussed later, Eq. (15) in Section 4.2.3). The 0 K apparent activation volumes and MSADs of the present alloys are then fitted with Eq. (8), solid red line in Fig. 5(d), from which *l* is deduced to be 1.9. This value means that on average, a dislocation segment will slip by 1.9*b* during a thermal activated jump in these concentrated solid-solution alloys. In comparison, *l* was estimated to be 5–4 in some Cu-based binaries with solute concentrations 5 ~ 11 at.% [44]. A possible reason for this difference is that the distance traveled by dislocations during an activated jump (proportional to *l*) is larger in dilute solid-solutions (such as the Cu-based binary alloys) than in concentrated solid-solutions (HEA and MEAs) because the distance between solutes that act as obstacles for the dislocation motion is larger in the former alloys.

**Figure 6.**
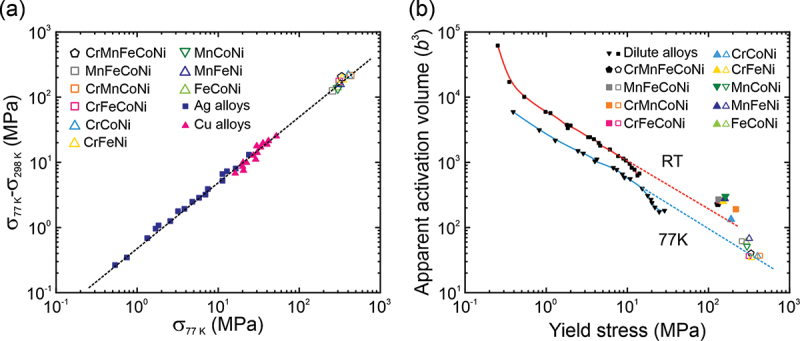
(a) Difference between the yield stress at 77 K and at room temperature plotted as a function of the yield stress at 77 K for dilute FCC solid-solutions [45], HEA and MEAs. (b) Apparent activation volumes at 77 K and room temperature plotted as a function of yield stress for FCC solid-solutions [45], HEA and MEAs.

According to the concept of ‘stress-equivalence’ proposed by Basinski et al. [45], the temperature and strain-rate dependences of CRSS correlate with the magnitude of solid solution hardening regardless of the type of solute and/or its concentration. As shown in Fig. 6(a) and 6(b), the concept of ‘stress-equivalence’ is roughly valid also for the quinary, quaternary and ternary equiatomic high- and medium-entropy alloys of the Cr-Mn-Fe-Co-Ni system. However, since some of the CRSS-temperature curves in Fig. 1(a) cross each other, and the apparent activation volumes in Fig. 6(b) do not all lie on a single line, ‘stress-equivalence’ is a relatively crude concept. Their conclusion that a single and common mechanism, independent of the type and concentration of solute, is responsible for solid solution strengthening needs further investigation.

### 4.2. CRSS at 0 K for FCC HEA and MEAs

The main strengthening mechanism in the present alloys is solid solution strengthening. In what follows, we estimate qualitatively the extent of solid solution strengthening (SSS) in the present HEA and MEAs using a few different models that have been proposed so far.

#### 4.2.1. SSS model of Varvenne et al.

Varvenne et al. [39] expressed the strengthening due to the elastic interaction between solutes and dislocations as

(9) $$\tau_{0}=0.051\alpha^{-1/3}\mu \left(T\right){\left(\frac{1+\nu }{1-\nu }\right)}^{4/3}f_{1c}{\left[\frac{\sum_{n}c_{n}\left(\Delta {\bar{V}}_{n}^{2}+\sigma_{\Delta V_{n}}^{2}\right)}{b^{6}}\right]}^{2/3}$$

where *α* is a constant in the equation for dislocation line tension $\Gamma \left(\Gamma =\alpha \mu b^{2}\right)$, *f*_1*c*_ is the minimized dislocation structural coefficient taken as 0.35 when the dissociation width is larger than 10*b*, Δ*V*_*n*_ is the misfit volume of solute element *n*, and $\sigma_{\Delta V_{n}}^{\;}$ is the standard deviation due to different local chemical and structural environments. The misfit volume Δ*V*_*n*_ is given by

(10) $$\Delta {\bar{V}}_{n}=\sum_{m}c_{m}\left[\frac{\partial {\bar{V}}_{\mathrm{alloy}}}{\partial c_{n}}\left|{\;}_{\bar{c}}\right.-\frac{\partial {\bar{V}}_{\mathrm{alloy}}}{\partial c_{m}}\left|{\;}_{\bar{c}}\right.\right]$$

**Figure 7.**
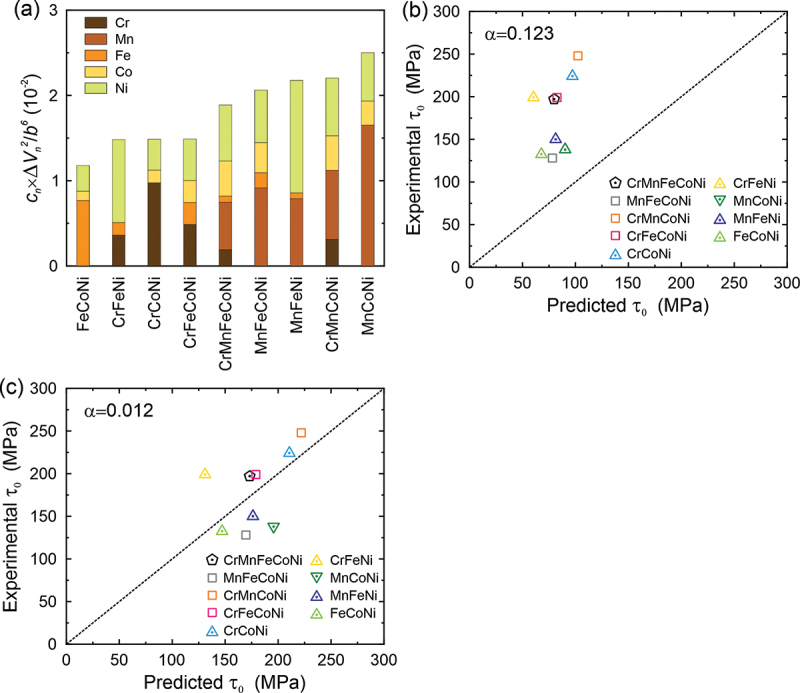
(a) The contribution of each constituent element to misfit volume in HEA and MEAs. The 0 K CRSS predicted by Eq. (9) with the recommended value of *α* = 0.123 (b) and optimized value of *α* = 0.012 (c).

Using the atomic volumes of Cr, Mn, Fe, Co and Ni in Varvenne et al. [39], the calculated $c_{n}\times \Delta V_{n}^{2}/b^{6}$of each element is shown in the bar chart of Fig. 7(a). Thus, according to the Varvenne model, Mn, Ni and Cr tend to contribute more to solid solution strengthening than Fe and Co but their relative contributions can vary significantly from alloy to alloy. Using these values and the recommended *α* of 0.123 [39], 0 K CRSSs were calculated and the results are plotted in Fig. 7(b) versus the experimental values. The 0 K CRSSs predicted using the Varvenne et al. [39] model fall significantly below the experimental values, although the order of their relative strengths is correctly predicted. A regression analysis was, therefore, performed to optimize the *α* value in their model. As shown in Fig. 7(c), the experimental 0 K CRSSs agree well with the model values if *α* = 0.012 is used instead of the recommended value of 0.123. This implies a significant reduction in the effective dislocation line tension and may indicate that dislocations with large curvatures are needed for their model to more accurately describe solid solution strengthening in the present HEA and MEAs.

#### 4.2.2. SSS model of Toda-Caraballo et al.

According to Toda-Caraballo et al. [21], solid solution hardening arises from the solute-dislocation interaction through the misfit in atomic size (*δ*_*n*_) and shear modulus ($\eta {{\;}^{\prime }}_{n}$) between the solute *n* and the imaginary solvent comprising the other elements, as described by the following equations,

(11) $$\delta_{n}=\frac{\mathrm{\partial a}}{\partial c_{n}}\frac{1}{a}\mathrm{and}\;\eta_{n}^{\prime }=\frac{\eta_{n}}{1+0.5\left|\eta_{n}\right|}\mathrm{with}\;\eta_{n}=\frac{\partial \mu }{\partial c_{n}}\frac{1}{\mu }$$

where *a* is the lattice parameter, and *μ* is the shear modulus. The CRSS at 0 K is then calculated to be

(12) $$\tau_{0}={\left(\sum_{n}B_{n}^{3/2}c_{n}\right)}^{2/3}\mathrm{with}\;B_{n}=\mu {\left(\eta_{n}^{\prime }{\;}^{2}+\varsigma^{2}\delta_{n}^{2}\right)}^{2/3}Z$$

where *Z* is a fitting parameter and *ς* is a parameter accounting for the difference in interaction energy between screw/edge dislocations and solutes. We adopted *ς* = 16 for edge-dislocation/solute interaction following earlier work [46]. According to Labusch [47], on the other hand, the 0 K CRSS $\tau_{0}^{L}$ is given by

(13) $$\tau_{0}^{L}=\frac{\varepsilon^{4/3}{\left({\eta_{n}^{\prime }}^{2}+\varsigma^{2}\delta_{n}^{2}\right)}^{2/3}d^{1/3}}{{\left(4\Gamma \right)}^{1/3}}c^{2/3}\mathrm{with}\;\varepsilon \propto \mathrm{\mu b}$$

where *d=βb* is the range of the interaction force (or dislocation core width). By comparing Eqs. (12) and (13), we obtained

(14) $$Z\propto {\left(\frac{\beta }{\alpha }\right)}^{1/3}$$

**Figure 8.**
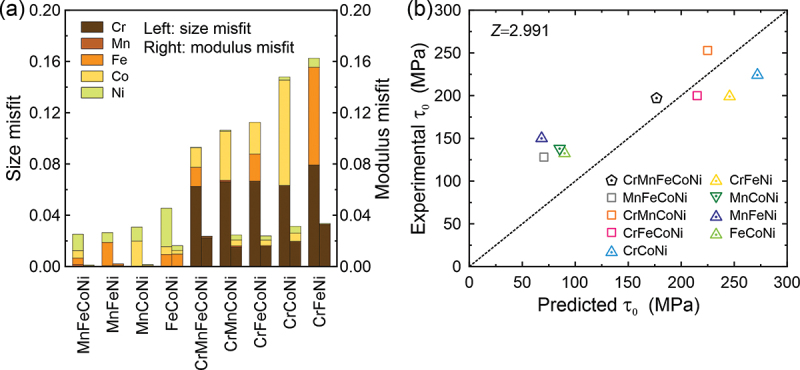
(a) Contributions from the size misfit term (left axis) and modulus misfit term (right axis) to the 0 K CRSS estimated with Eq. (11). (b) 0 K CRSS predicted with Eq. (12) and *Z* = 2.991 for the equiatomic HEA and MEAs.

*β* is reported to vary from 0.5 to 1.5 for edge dislocations in Cu and Al by Seeger et al. [48]. Wu et al. [15] found that *β* = 1 best fits the temperature dependence of yield stress in polycrystals of the Cr-Mn-Fe-Co-Ni HEA and its FCC derivative MEAs. Therefore, the difference in *Z* would appear to be mainly due to the difference in the dislocation line tension coefficient *α*. We then estimated the contribution of each element to the size misfit and modulus misfit terms in the present HEA and MEAs, as shown in Fig. 8(a). A notable feature is that the size misfit term dominates in these alloys. In contrast to the model of Varvenne et al. [39], this model predicts that Cr contributes significantly to the size misfit term with a non-negligible contribution also from Co. In addition, Cr is found to contribute substantially to the modulus misfit term. The fitting parameter *Z*_HEA_ = 2.991 is then determined by a regression analysis of the calculated and experimental 0 K CRSSs, as shown in Fig. 8(b). This value is much larger than the *Z*_dil_ values determined for FCC solid-solution alloys (1.9, 1.4 and 0.8 for dilute Ag, Au and Cu binary solid-solution alloys, respectively [46]). Assuming that *β* = 1 (or at least constant) in Eq. 14, the higher Z value for the HEA and MEAs suggest that a smaller line tension coefficient (*α*) is needed to reproduce the experimental 0 K CRSSs using the model of Toda-Caraballo et al. [21]. It appears, therefore, that both models, Varvenne et al. [39] and Toda-Caraballo et al. [21] require a low value of line tension to reproduce experimentally measured CRSSs. This is an intriguing result and its physical implications are worth further exploration.

#### 4.2.3. Mean-square atomic displacement (MSAD)

**Figure 9.**
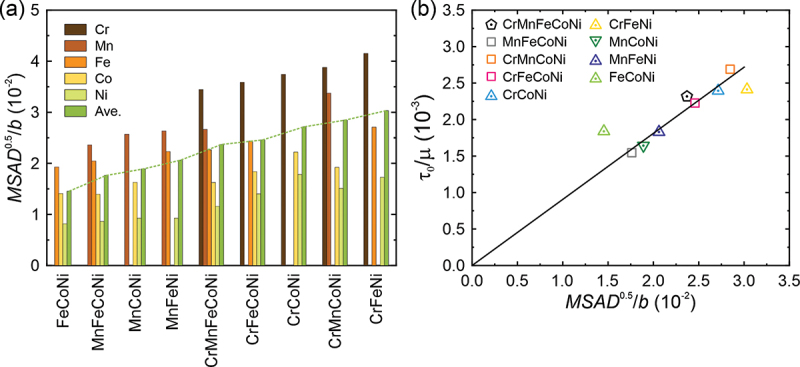
(a) Square root of MSAD for each of the constituent elements and averaged values for the HEA and MEAs. (b) Relation between the experimental 0 K CRSS (normalized by the shear modulus) and square root of MSAD (normalized by the dislocation Burgers vector) for the present HEA and MEAs.

Mean-square atomic displacement (MSAD), which is a measure of the average displacements of the constituent atoms from the ideal FCC lattice positions, has been shown to correlate with the 0 K CRSS of FCC HEAs and MEAs [16,22]. Fig. 9(a) charts the square root of MSAD normalized by the Burgers vector for each of the constituent elements along with their averaged values for the different equiatomic alloys. The contribution to the averaged MSAD follows the order Cr>Mn>Fe>Co>Ni, that is, alloys containing Cr have higher MSADs. Cr also plays an important role in strengthening these alloys, as shown previously in Fig. 8(a). We, therefore, plotted the 0 K CRSS normalized by shear modulus *μ* as a function of the square root of the averaged MSAD normalized by the magnitude of Burges vector in Fig. 9(b). The 0 K shear moduli were obtained by extrapolating the temperature-dependent shear moduli reported by Laplanche et al. [49,50]. A good linear relationship spanning a relatively wide range of compositions is observed, indicating that MSAD is a reasonable scaling factor to predict solid solution hardening in FCC HEA and MEAs. The plotted data points for the equiatomic Cr-Fe-Ni and Fe-Co-Ni MEAs (which lie off the line) may be affected by their magnetic properties, because we calculated MSAD at 0 K without considering the magnetic moment (i.e. in the non-magnetic state), while the experimental CRSS at 0 K is obtained by extrapolation from higher temperatures where magnetic properties play a role. A paramagnetic-antiferromagnetic transition is reported to occur in the Cr-Fe-Ni MEA [51], while a paramagnetic-ferromagnetic transition is observed in the Fe-Co-Ni MEA [50]. This may explain the relatively large deviation of these two alloys from the linear MSAD fit.

The linear relation between the 0 K CRSSs and MSADs when normalized, respectively, by the shear modulus and the magnitude of Burgers vector can be expressed as,

(15) $$\frac{\tau_{0}}{\mu }=C\frac{\mathrm{MSA}D^{1/2}}{b}$$

For the present equiatomic HEA and MEAs, the constant *C* is determined to be 0.091. This is very close to the value (*C* = 0.094) determined for non-equiatomic Cr-Mn-Fe-Co-Ni HEAs [9], indicating that MSAD is a reasonable scaling factor to predict solid solution hardening in FCC HEA and MEAs, regardless of the number of constituent elements.

### 4.3. Dislocation inertial effects

The dislocation inertial effects have been attributed to the decrease in the frictional (damping) coefficient, *B*. In general, the frictional coefficient is believed to comprise three terms, *B*_e_, *B*_p_, and *B*_r_ due to conduction electrons, phonons and radiation of acoustic waves, respectively. *B*_e_ is temperature-independent in the ‘normal’ state but it drops substantially in the superconducting state below the transition temperature. This has been reported to be one of the reasons for the inertial effects in some FCC pure metals and alloys below 1–2 K [37]. However, as the lowest temperature employed in the present study was only 9 K, this effect can be ruled out here. *B*_r_ is usually estimated to be several tenths of *B*_e_ and is temperature-independent as suggested by Schwarz et al. [52]; it can, therefore, also be ignored. That leaves a decrease in *B*_p_ at cryogenic temperatures as the most plausible reason for the inertial effects observed in the present alloys. *B*_p_ can be calculated at low temperatures by the following equation [53]

(16) $$B_{P}=\frac{14.4k_{B}T\omega_{D}^{2}}{\pi^{2}\upsilon_{s}^{3}}{\left(\frac{T}{\theta_{D}}\right)}^{2}$$

where *k*_B_ is the Boltzmann constant, *ω*_D_ the Debye frequency, *θ*_D_ the Debye temperature, and *υ*_s_ the sound velocity. For FCC crystals, *θ*_D_ and *ω*_D_ are given by [54]

(17) $$\theta_{D}=\frac{h}{k_{B}}{\left(\frac{6\pi^{2}\rho }{M}\right)}^{1/3}\upsilon_{s};\omega_{D}=\frac{k_{B}\theta_{D}}{h}$$

where *ρ* is the alloy density, *M* the atomic weight, and *h* the Planck constant. *υ*_s_ can be estimated by [54]

(18) $$\upsilon_{s}=f\left(\nu \right)\sqrt{\frac{K}{\rho }}$$

where *K* is the bulk modulus, *ν* is the Poisson ratio and *f*(*ν*) is

(19) $$f\left(\nu \right)={\left\{\frac{1}{3}\left\{{\left[\frac{1+\nu }{3\left(1-\nu \right)}\right]}^{3/2}+2{\left[\frac{2\left(1+\nu \right)}{3\left(1-2\nu \right)}\right]}^{3/2}\right\}\right\}}^{-1/3}$$

**Figure 10.**
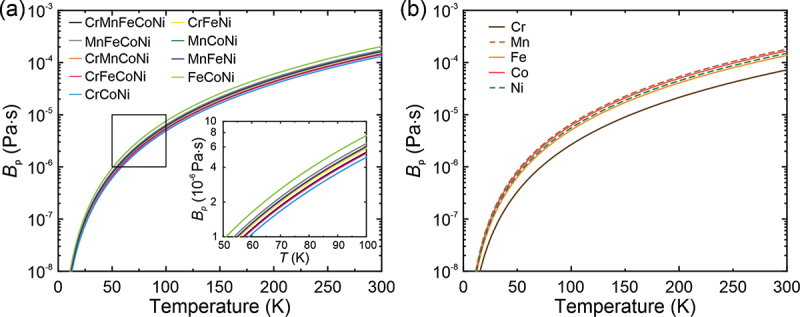
Estimated temperature dependence of the frictional drag coefficient due to phonon scattering: (a) the present HEA and MEAs and (b) the five constituent elements.

Using the elastic constants reported by Laplanche et al. [50], the phonon drag coefficient *B*_p_ was estimated as a function of temperature below 300 K, and the results are shown in Fig. 10(a). *B*_p_ decreases significantly with decreasing temperature especially below 100 K. Schwarz et al. [52] proposed that the critical drag coefficient (*B*_c_) for the manifestation of dislocation inertial effects is

(20) $$B_{c}=\frac{6f_{0}{\left(\Gamma m\right)}^{1/2}}{b}{\left[\frac{c/f_{0}}{1+3\lambda {\left(c/f_{0}\right)}^{1/2}}\right]}^{1/2}$$

where *Γ* is the line tension of dislocations, *m* is the dislocation mass per unit length, *f*_0_ is the normalized strength of obstacles, *c* is the solute concentration, and *λ* is a constant (2.5). For the equiatomic Cr-Mn-Fe-Co-Ni HEA, since all constituents are 20 at.% each, *c* = 0.2 and *B*_c_ can be estimated to be about 1.5×10^−5^ Pa·s. Given that the temperature-independent *B*_e_ ~1×10^−5^ Pa·s and *B*_r_=*B*_c_/16 [55,56], the critical *B*_pc_ at which the dislocation inertial effects may be expected to occur is ~ 4×10^−6^ Pa·s. Consistent with this, *B*_p_ decreases to ~ 4×10^−6^ Pa·s at around 90 K in the Cr-Mn-Fe-Co-Ni HEA (Fig. 10(a)), which is close to the temperature (77 K) below which a dulling of the temperature dependence and strain-rate sensitivity of CRSS is observed.

The phonon drag coefficients (*B*_p_) of the elements Cr, Mn, Fe, Co, and Ni are plotted as a function of temperature in Fig. 10(b), from which it can be seen that Cr and Mn exhibit the lowest and highest *B*_p_, respectively. Assuming a rule of mixtures, this suggests *B*_p_ may decrease with increasing Cr content and increase with increasing Mn content in alloys of the Cr-Mn-Fe-Co-Ni system. Therefore, the critical phonon-drag coefficient *B*_pc_ (at which inertial effects are manifested) occurs at higher temperatures in alloys containing Cr than in those containing Mn. This may also be one of the reasons for the fact that the degree of inertial effects becomes less prominent in Cu-Al alloys with increasing Al content [35], since Al has a higher phonon drag coefficient (20×10^−5^ Pa·s) than Cu (1.6×10^−5^ Pa·s) at 100 K.

### 4.4. Portevin-Le Chatelier effect

**Figure 11.**
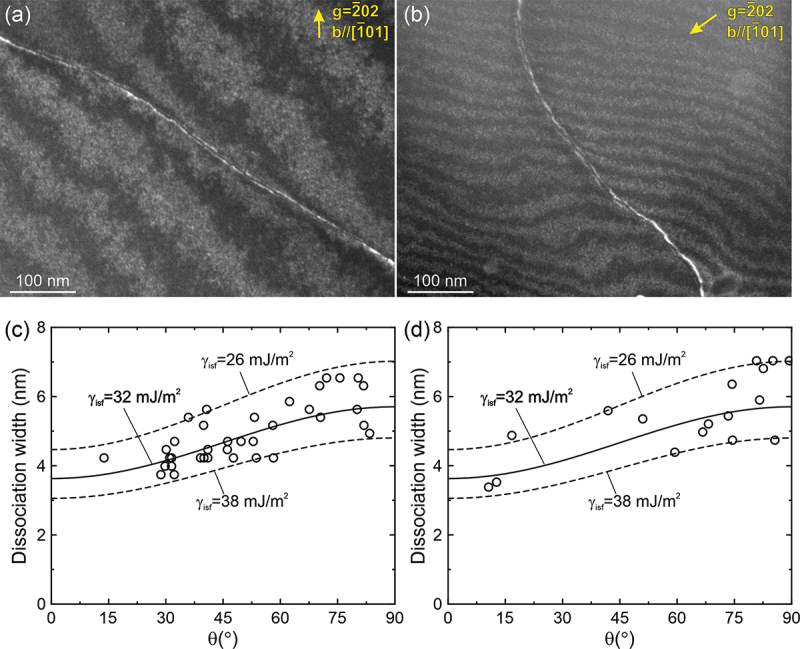
Weak-beam TEM images of two-coupled partials introduced in [123]-oriented single crystals of the equiatomic Cr-Fe-Ni MEA deformed in compression to about 3% plastic strain at (a) room temperature and (b) 1173 K. Dissociation widths of coupled-partial dislocations plotted as a function of angle between the Burgers vector and dislocation line are shown in (c) and (d) for room temperature and 1173 K, respectively.

Portevin-Le Chatelier (PL) effect is believed to arise from the interaction between solute atoms and moving dislocations in solid-solution alloys. The chemical attraction of solute atoms to stacking faults (SFs) in FCC alloys, the Suzuki effect, is usually believed to be responsible for the occurrence of PL effect. The postulated mechanism involves solutes that decrease the energy of SFs segregating there and hindering dislocation motion. Accordingly, Cr and Co, which decrease SF energy [9], must enhance the PL effect in the present alloys. To check whether the dissociation width between two-coupled partial dislocations increases or not, [$\bar{1}$23]-oriented single crystals of the equiatomic Cr-Fe-Ni MEA were deformed in compression to about 3% plastic strain at room temperature and 1173 K (the peak temperature of yield strength increase at which serrations are intensively observed). After compression testing at 1173 K, the optical furnace was immediately turned off and removed from the testing machine so that the specimens could be cooled as quickly as possible (down to below 473 K within ~ 15 s) in vacuum. We wish to make it clear that we did not aim to measure the SFE at 1173 K (this should be done by in-situ observations) but to investigate whether Suzuki effect occurs or not when the specimen is deformed at 1173 K (the peak temperature of yield strength increase at which serrations are intensively observed) in the Cr-Fe-Ni MEA. We believe that if the Suzuki segregation occurs during the high temperature deformation, most of the segregated solutes in the SFs cannot de-segregate and diffuse back into the FCC matrix due to the energy penalty of de-segregation and the quick decay in atomic diffusion during the above cooling process. After deformation at both room temperature (Fig. 11(a)) and 1173 K (Fig. 11(b)), dislocations are clearly observed to dissociate into two Shockley partial dislocations. The dissociation widths are measured in Fig. 11(c) and 11(d) as a function of angle between the Burgers vector and dislocation line. No particular variation in dissociation width at the same character is observed even after deformation at the peak temperature 1173 K when compared with that at room temperature. Consequently, the estimated stacking fault energy fitted with the equation based on isotropic theory 32 ± 6 mJ/m^2^ is essentially the same as that obtained from the specimen deformed at room temperature, indicating that no significant segregation of Cr to the stacking fault occurs during serrated flow at high temperatures. This may suggest that the Suzuki effect (i.e. segregation of Cr (and Co) to the stacking fault) is not the origin for the PL effect. On the other hand, the increase in CRSS is found to be positively correlated with Mn and/or Fe content (Fig. 3(c), 3(d) and 3(e)), which are known to increase rather than decrease the SF energy of these alloys. In addition, Mn is reported to be the fastest diffuser in the Cr-Mn-Fe-Co-Ni HEA [57–59]. These features suggest that the Cottrell effect through the elastic interaction between solutes and dislocations, instead of the Suzuki effect, may be causing the PL effect, although the Suzuki effect cannot be summarily ruled out in the absence of direct evidence. In the following, we estimate the magnitude of dragging stress for dislocations assuming that the Cottrell effect is responsible for the PL effect. According to Sakamoto [60], the dragging stress (*τ*_d_) due to the Cottrell atmosphere in highly concentrated solid-solutions is given by

(21) $$\tau_{d}=\frac{\Lambda c\left(1-c\right)A^{2}}{k_{B}Ta_{i}b\Omega }{\left[{\left(\frac{\pi }{2}\ln\frac{\Lambda +1.2}{\Lambda }\right)}^{-1}+0.29\Lambda +0.16\Lambda^{2}\right]}^{-1}$$

where *A*=(1+*ν*)*μb*Δ*V*_*n*_/3*π*(1-*v*), Δ*V*_*n*_ is the misfit volume between solute and solvent atoms, *a*_*i*_ (= *b*) is the lattice constant of the imaginary lattice moving with the gliding dislocation, $\Omega =b^{3}/\sqrt{2}$ is the volume of the lattice, *Λ*=*υa*_*i*_/*D*, *υ* is the dislocation motion velocity, and *D* is the diffusion coefficient. Dislocation velocity can be estimated from the Orowan equation ($\dot{\gamma }=\rho_{m}\mathrm{\upsilon b}$, where $\dot{\gamma }$ is the shear strain rate, and *ρ*_m_ is the mobile dislocation density). The calculated values of *τ*_d_ for the present HEA and MEAs are tabulated in Table 4 together with the experimental Δ*τ* values. The experimental Δ*τ* values are plotted in Fig. S4 as a function of the calculated *τ*_d_ values, showing a nice agreement between the two values except for an underestimation for the Mn-Fe-Ni MEA. Note that not just the individual element concentrations but also the specific combination of constituent elements affects the dragging force, as the misfit volume between solute and solvent atoms varies from alloy to alloy even for a particular solute element. For example, the Mn and Fe concentrations are higher in equiatomic Mn-Fe-Ni than in equiatomic Mn-Co-Ni, but the misfit volume between Mn and the effective matrix atoms is much smaller in the former alloy based on the results of Varvenne et al. [39], resulting in a lower calculated *τ*_d_ for the Mn-Fe-Ni alloy. Additionally, we used the diffusion coefficient *D* of Mn and Fe obtained in the equiatomic Cr-Mn-Fe-Co-Ni HEA to calculate the *τ*_d_ values for the Mn-Fe-Co-Ni, Cr-Mn-Co-Ni, Mn-Fe-Ni and Mn-Co-Ni MEAs due to the lack of experimental diffusion data for these MEAs from literatures. However, the diffusion behavior of a particular element usually varies with the matrix. In particular, Mn is reported to diffuse more slowly in Fe than in Co [57]. This implies that Mn may diffuse more slowly in the Mn-Fe-Ni MEA than in the Mn-Co-Ni MEA. According to Eq. (20), the calculated *τ*_d_ is negatively correlated with the diffusion coefficient *D*, thus assuming a high diffusion coefficient will result in a lower calculated *τ*_d_. This may be also one of the reasons for the underestimation of the *τ*_d_ value in the Mn-Fe-Ni MEA. In short, the model manifests itself the capability of semi-quantifying the *τ*_d_ value due to PL effect in substitutional solid-solutions but its accuracy in the prediction depends much on materials parameters, especially the misfit volume and diffusion coefficient, both of which differ significantly from research group to research group in the studies of HEA and MEAs. We then tend to believe that the Cottrell effect is supposed to be, at least, one of the main reasons for the PL effect in the present HEA and MEAs. Note that the possibility of the occurrence of Suzuki effect is not denied without direct evidence, and this is currently under investigation by in-situ TEM observation and atom probe tomography in our group.

## 5. Conclusions

The temperature-dependent plastic deformation behaviors of [$\bar{1}$23]-oriented single crystals of quaternary and ternary equiatomic MEAs of the Cr-Mn-Fe-Co-Ni system were investigated in compression in the temperature range 9 K to 1373 K and compared to earlier results obtained on single crystals of the equiatomic HEA Cr-Mn-Fe-Co-Ni, and the equiatomic MEAs Cr-Fe-Co-Ni and Cr-Co-Ni. The following conclusions were reached.

1. All alloys exhibit similarly shaped CRSS vs. *T* curves, although the magnitude of CRSS at any given temperature varies significantly from alloy to alloy.
2. CRSSs increase rapidly with decreasing temperature below room temperature accompanied by a dulling of the temperature dependence of CRSS at temperatures below 77 K due to dislocation inertial effects arising from the decrease in phonon drag coefficient. Cr has the strongest effect on this dulling and Mn the weakest.
3. CRSSs increase moderately at high temperatures above 673 K ~ 873 K due to the Portevin-Le Chatelier (PL) effect through elastic dislocation-solute attraction. The CRSS increase due to the PL effect is found to be greatest for Mn and Fe.
4. The apparent activation volumes for deformation of the present alloys can be well described by the conventional theory of thermally activated dislocation motion in dilute FCC alloys. Those alloys containing Cr exhibit stronger dislocation glide resistance, and thus lower apparent activation volumes; consistent with this, they exhibit stronger temperature dependences of CRSS compared to alloys without Cr. The stress-equivalence originally proposed for binary FCC alloys appears to hold for the present MEAs, although only in a crude sense.
5. The CRSSs at 0 K show good linear correlation with the square root of the averaged MSADs of the equiatomic Cr-Mn-Fe-Co-Ni HEA and the equiatomic, quaternary and ternary MEAs based on the same constituent elements. This suggests that MSAD is a good scaling factor to predict CRSS in highly concentrated solid-solution alloys.

## Supplementary Material

Supplemental Material
