# Supplementary material for: Analysis of the temperature-dependent plastic deformation of single crystals of quinary, quaternary and ternary equiatomic high- and medium-entropy alloys of the Cr-Mn-Fe-Co-Ni system
Source: Sci Technol Adv Mater. 2024 Jul 8;25(1):2376524. doi: 10.1080/14686996.2024.2376524 (PMC11302478; doi:10.1080/14686996.2024.2376524)
Supplement: Supplemental Material [file TSTA_A_2376524_SM9024.pdf]

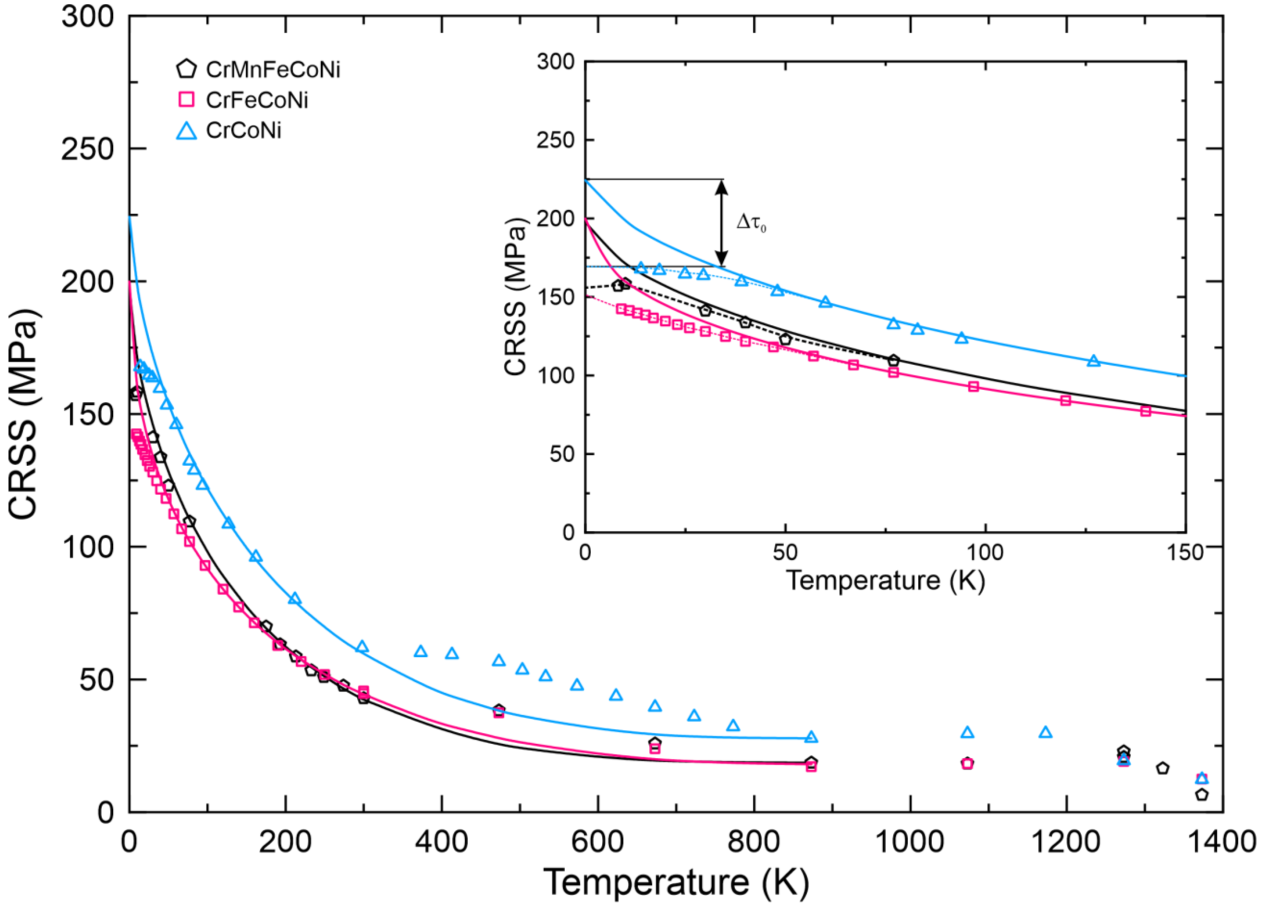

Fig. S1. Temperature dependence of CRSS for [$\bar{1}$23]-oriented single-crystals of equiatomic Cr-Mn-Fe-Co-Ni HEA [18], Cr-Fe-Co-Ni [20] and Cr-Co-Ni MEAs [19] from 9 K to 1373 K in compression at a strain rate of 1×10^{-4} s^{-1}. The inset shows a magnified view of the low-temperature region and an example of how the dulling of the CRSS ($\Delta\tau_0$) due to inertial effects is measured.

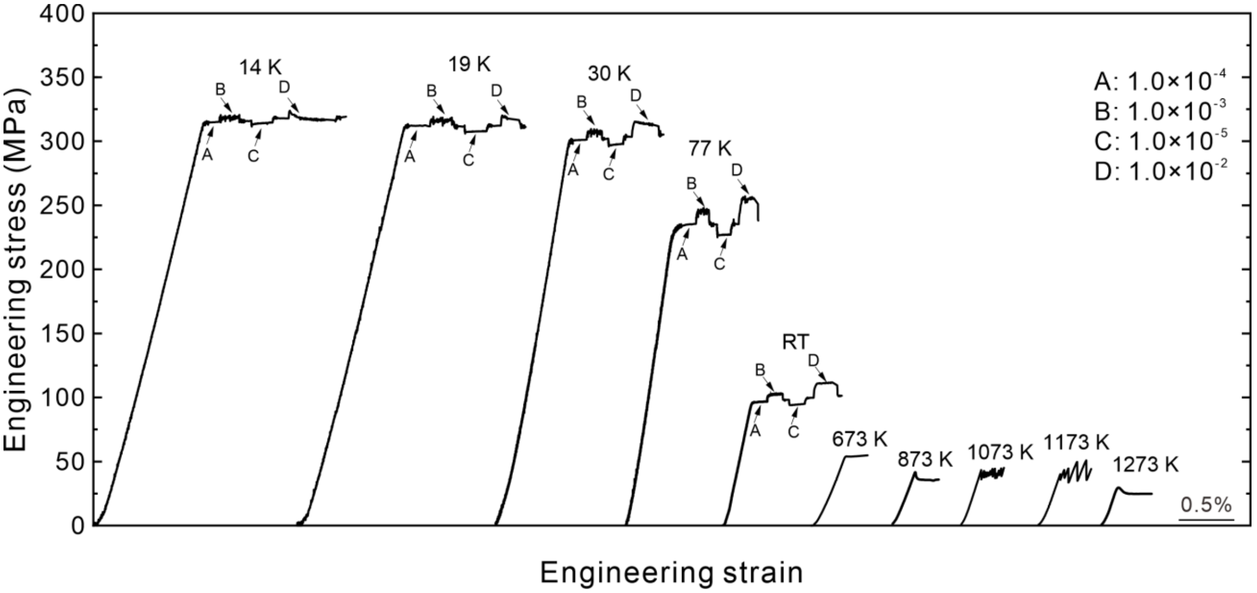

Fig. S2. Engineering stress-strain curves for [$\bar{1}$23]-oriented single crystals of the equiatomic Cr-Fe-Ni deformed in compression at selected temperatures from 14 K to 1273 K.

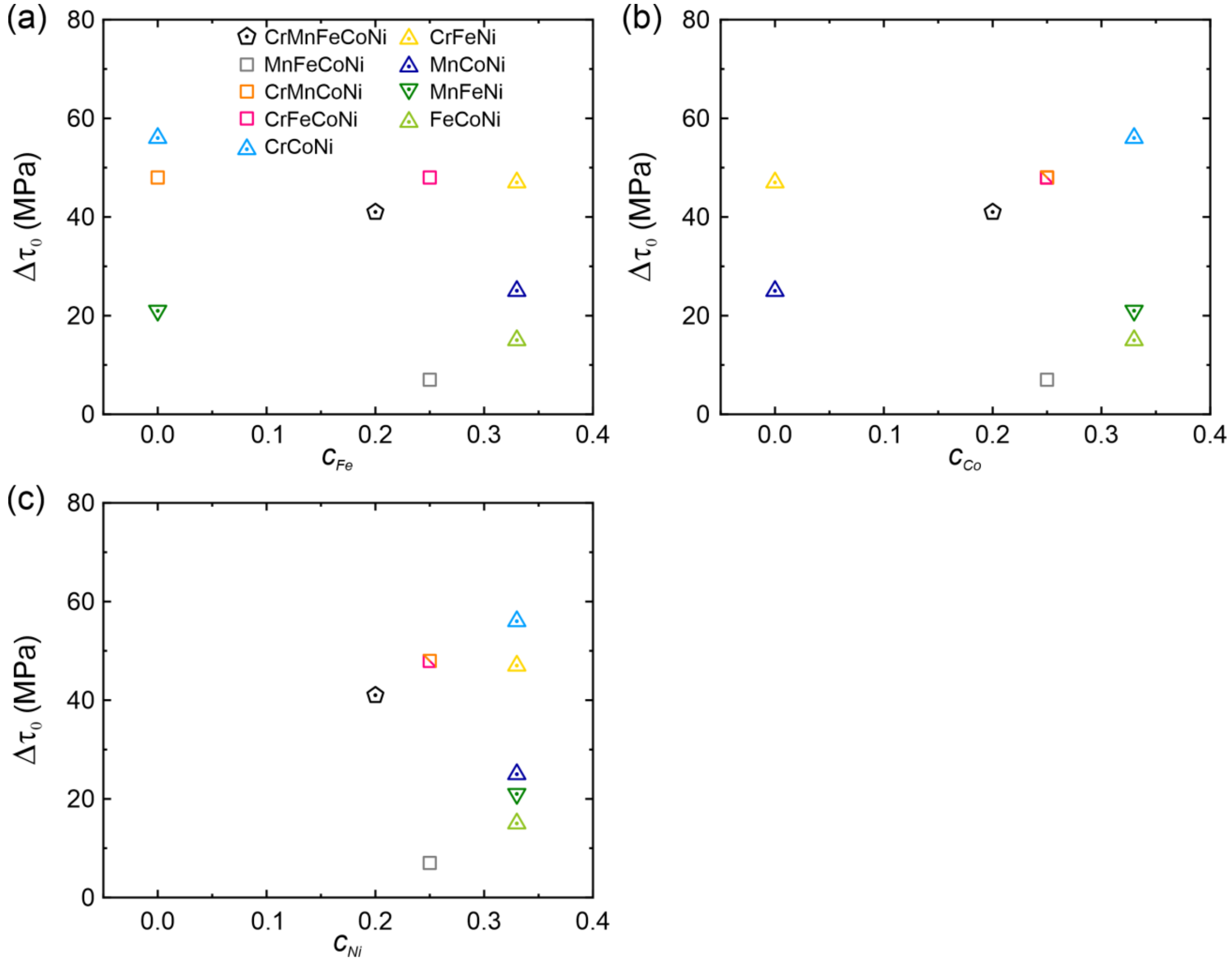

Fig. S3. The dulling of the CRSS ($\Delta\tau_0$) is plotted as a function of Fe content in (a), Co content in (b) and Ni content in (c).

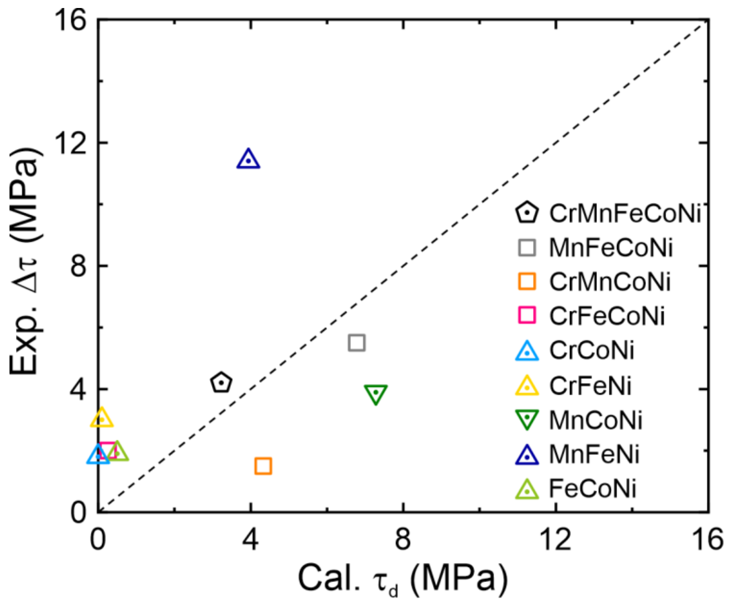

Fig. S4. The increase in CRSS obtained by experiments (Exp. $\Delta\tau$) at elevated temperatures plotted as a function of the calculated value (Cal. τ_d) by Eq. (21).
